# A synthetic methylotrophic *Escherichia coli* as a chassis for bioproduction from methanol

**DOI:** 10.1038/s41929-024-01137-0

**Published:** 2024-04-23

**Authors:** Michael A. Reiter, Timothy Bradley, Lars A. Büchel, Philipp Keller, Emese Hegedis, Thomas Gassler, Julia A. Vorholt

**Affiliations:** Institute of Microbiology, Department of Biology, ETH Zurich, Zurich, Switzerland

**Keywords:** Metabolic engineering, Industrial microbiology, Biosynthesis

## Abstract

Methanol synthesized from captured greenhouse gases is an emerging renewable feedstock with great potential for bioproduction. Recent research has raised the prospect of methanol bioconversion to value-added products using synthetic methylotrophic *Escherichia coli*, as its metabolism can be rewired to enable growth solely on the reduced one-carbon compound. Here we describe the generation of an *E. coli* strain that grows on methanol at a doubling time of 4.3 h—comparable to many natural methylotrophs. To establish bioproduction from methanol using this synthetic chassis, we demonstrate biosynthesis from four metabolic nodes from which numerous bioproducts can be derived: lactic acid from pyruvate, polyhydroxybutyrate from acetyl coenzyme A, itaconic acid from the tricarboxylic acid cycle and *p*-aminobenzoic acid from the chorismate pathway. In a step towards carbon-negative chemicals and valorizing greenhouse gases, our work brings synthetic methylotrophy in *E. coli* within reach of industrial applications.

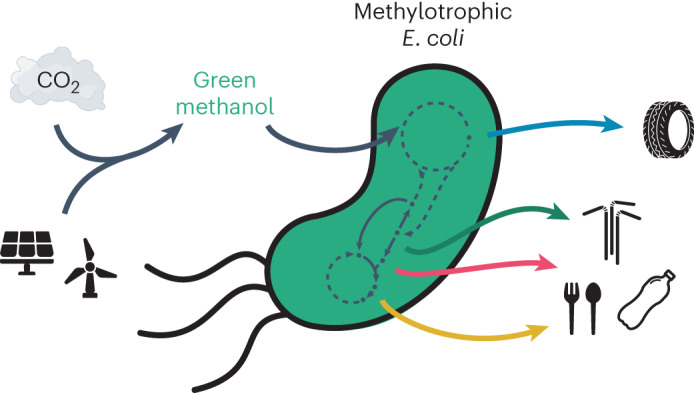

## Abstract (web summary)

Synthetic methylotrophic organisms provide potential for valorization of greenhouse gas-derived methanol. Here an *Escherichia coli* strain is generated that reaches a similar growth rate on methanol to many natural methylotrophs and is capable of producing chemicals from this carbon source.

## Main

Anthropogenic climate change is one of the most pressing problems of the twenty-first century. Under the Paris Agreement, nations around the world have committed to reducing greenhouse gas emissions to net-zero by 2050 (ref. ^1^). The carbon-intensive^2^ and hard-to-abate^3^ chemical sector poses a particular challenge. In the future, the bioconversion of green methanol may enable the economical production of carbon-negative chemicals. The substrate, green methanol, can be produced sustainably from biomass (bio-methanol) or synthesized from the greenhouse gases carbon dioxide or methane (e-methanol)^4^. In contrast to conventional biotechnology, green methanol-fuelled bioprocesses are independent of plant-derived sugars and, consequently, already severely strained agricultural resources^5–10^. Commercial green methanol production plants are now coming online and the annual capacity is expected to grow to about eight million metric tons up to 2027 (ref. ^11^).

The efficient bioconversion of methanol requires a suitable microbial production chassis. Natural methanol-utilizing organisms, that is, methylotrophs^12^, have been explored since the 1960s and 1970s, initially for single-cell protein production^13^ and more recently for chemical biosynthesis^14–17^. However, they have proved difficult to use for large-scale industrial applications due to a lack of advanced genetic tools and the absence of extensive bioprocessing experience.

To overcome these limitations, great effort has been expended over the past decade to convert *Escherichia coli* into a synthetic methylotroph^18–26^, among other model organisms frequently used in industrial applications^27–31^. Engineering projects in *E. coli* benefit from a vast ecosystem that spans academia and industry. Furthermore, *E. coli* has proved to be a viable biotechnology workhorse in numerous commercial fermentation processes^32–42^. Imparting *E. coli* with the additional ability to grow on methanol would enable the use of green methanol and, thus, greenhouse gas valorization to bioproducts.

**Fig. 1:**
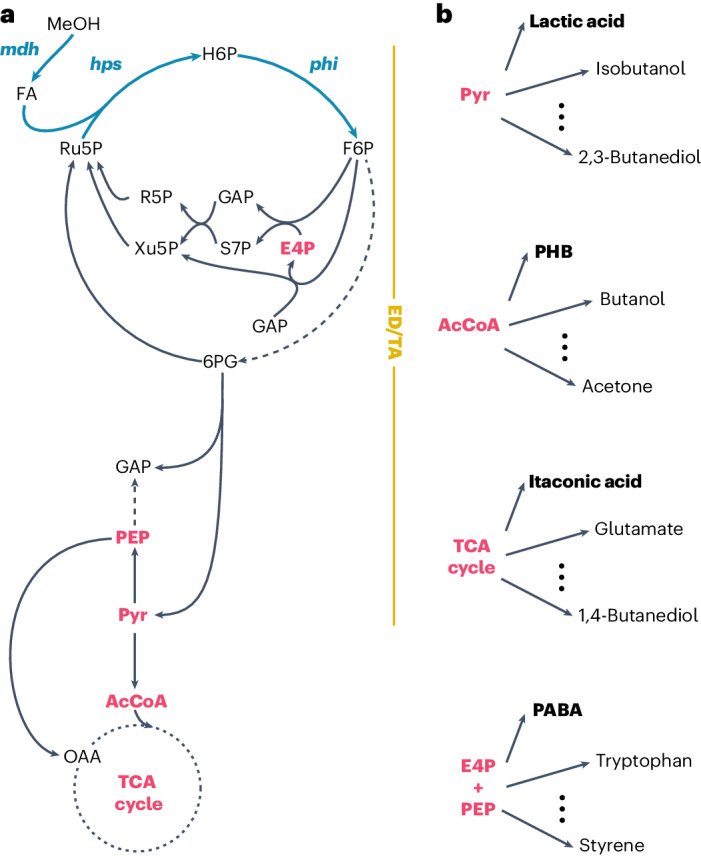
Bioproduction using synthetic methylotrophy in *E. coli*. **a**, Methanol metabolism in methylotrophic *E. coli*. To enable methanol assimilation, three genes are expressed heterologously in *E. coli*: a methanol dehydrogenase (encoded by *mdh*), a 3-hexulose 6-phosphate synthase (*hps*) and a 6-phospho 3-hexuloisomerase (*phi*). The synthetic methylotrophic *E. coli* strain operates the Entner–Doudoroff/transaldolase (ED/TA) variant of the RuMP cycle. Depicted is the carbon flow from methanol (MeOH) via the RuMP cycle to lower metabolism^44^. Dashed lines indicate multiple reactions. FA, formaldehyde; H6P, 3-hexulose 6-phosphate; F6P, fructose 6-phosphate; 6PG, 6-phosphogluconate; Ru5P, ribulose 5-phosphate; E4P, erythrose 4-phosphate; GAP, glyceraldehyde 3-phosphate; S7P, sedoheptulose 7-phosphate; R5P, ribose 5-phosphate; Xu5P, xylulose 5-phosphate; PEP, phosphoenolpyruvate; Pyr, pyruvate; OAA, oxaloacetate. **b**, Four metabolic nodes and examples of derived commodity as well as speciality chemicals^42,49^. Throughout, compounds produced in this study are shown in bold.

Recently, Kim and co-workers reported growth on methanol by an *E. coli* strain via the reductive glycine pathway (doubling time (*T*_d_) ≈ 54 h)^43^. We^44^ and others^45^ engineered and evolved the highly efficient ribulose monophosphate (RuMP) cycle^46,47^ in *E. coli* (Fig. 1a), which resulted in strains growing at doubling times of around 8 h. However, the engineering of these synthetic methylotrophs for bioproduction from methanol has not yet been shown.

Here we establish synthetic methylotrophy as a new mode for bioproduction from methanol. We first improved growth on methanol and achieved a doubling time of 4.3 h. This is faster than the natural model methylotroph *Bacillus methanolicus* (*T*_d_ = 5 h) at 37 °C (ref. ^48^). Building on top of this synthetic *E. coli* strain, we demonstrate production from key metabolic nodes, which serve as starting points to a plethora of bioproducts^42,49^: lactic acid from pyruvate, polyhydroxybutyrate (PHB) from acetyl coenzyme A (AcCoA), itaconic acid from the tricarboxylic acid (TCA) cycle and *p*-aminobenzoic acid (PABA) from the chorismate pathway (Fig. 1b). Finally, we show growth of the synthetic methylotroph in a fed-batch bioreactor on methanol to an optical density (at 600 nm; OD_600_) of 100 and enhanced itaconic acid production, which reached 1 g l^−1^. This study highlights the potential of synthetic methylotrophy in *E. coli* and lays the foundation for future industrial bioconversion of methanol.

## Results

### Methylotrophic growth of *E. coli* with a 4.3 h doubling time

In a previous study, we demonstrated the methylotrophic growth of *E. coli*^44^. Synthetic methylotrophy was achieved after evolving a methanol-dependent strain for about 250 generations via continuous chemostat culture. To improve growth on methanol, the methylotrophic population was propagated under a serial dilution evolution regime. From this experiment, a methylotrophic reference strain was isolated after 255 generations (MEcoli_ref_1), which exhibited improved growth (*T*_d_ = 8.1 ± 0.4 h (mean ± s.d.)) compared with the initial population after chemostat evolution (*T*_d_ ≈ 60 h)^44^.

**Fig. 2:**
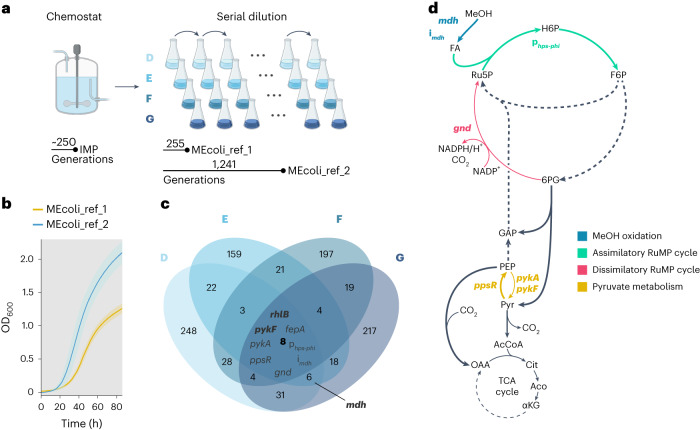
Growth of MEcoli_ref_2 and its genetic adaptations. **a**, Generation of improved methylotrophic *E. coli* via serial dilution evolution. After methylotrophic growth was achieved during chemostat evolution for approximately 250 generations^44^, the initial methylotrophic population (IMP) was split into four replicate lines (D, E, F and G) that were subsequently propagated through serial dilution. Methylotrophic reference strains were isolated after 255 (MEcoli_ref_1)^44^ and 1,241 generations (MEcoli_ref_2) from replicate line D. **b**, Growth phenotypes of MEcoli_ref_1 and MEcoli_ref_2 in minimal medium supplemented with 500 mM methanol. Growth was assayed using a microplate reader by measuring the absorbance at 600 nm. The absorbance values were converted to OD_600_. The curves denote the mean values of three technical replicates, and the shaded areas denote the standard deviation error bands around the means. **c**, Venn diagram of mutational overlap of populations after 1,205 (D), 1,130 (E), 1,118 (F) and 1,119 (G) generations under serial dilution. Mutations present in the starter population used for the inoculation of the four serial dilution lines were not taken into account. Mutations were grouped at the gene or intergenic region level (for example, the gene symbol *pykA* aggregates four different mutations). Groups that acquired mutations that are present in MEcoli_ref_2 but not in MEcoli_ref_1 are highlighted in bold. *rhlB*, gene encoding ATP-dependent RNA helicase; *pykF*, gene encoding pyruvate kinase 1; *fepA*, gene encoding ferric enterobactin outer membrane transporter; *pykA*, gene encoding pyruvate kinase 2; p_*hps-phi*_, promoter region of the *hps-phi* operon; *ppsR*, gene encoding phosphoenolpyruvate synthetase regulatory protein; i_*mdh*_, intergenic region of the methanol dehydrogenase-encoding plasmid; *gnd*, gene encoding 6-phosphogluconate dehydrogenase. **d**, Repeatedly mutated functional units in the context of methanol metabolism. Coloured arrows and genetic element names indicate the associated functional unit. Thin lines depict reactions predicted by parsimonious flux balance analysis to carry low (that is, less than 10% of incoming, *gnd*) or no flux (*pykA*, *pykF*, part of the TCA cycle) (Supplementary Data Table 5). Dashed lines indicate more than one reaction. NADPH, reduced nicotinamide adenine dinucleotide phosphate; NADP^+^, nicotinamide adenine dinucleotide phosphate; Cit, citrate; Aco, *cis*-aconitate; αKG, α-ketoglutarate. Source data

**Extended Data Fig. 1:**
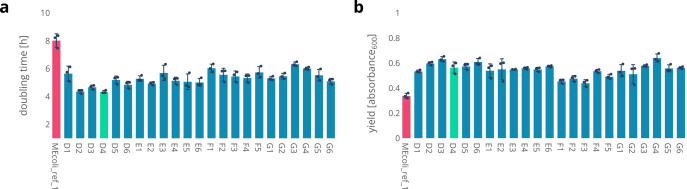
Growth phenotype of isolate clones. Letters in sample names indicate the replicate line the sample was isolated from. Clones were isolated from replicate lines after 1241 (line D), 1236 (E), 1218 (F) and 1230 (G) generations. The original methylotrophic reference strain, MEcoli_ref_1 is shown in pink. Sample D4, shown in green, was used as reference strain (MEcoli_ref_2) for all other experiments in this study. **a** Doubling times **b** Maximum measured optical density. Growth phenotype raw data is provided in Supplementary Data Table 16. Data are represented as mean values ± S.D. of n = 3 technical replicates. Source data

In the present study, we continued the serial dilution evolution experiment (four replicate lines D, E, F and G; Fig. 2a and Supplementary Data Table 1). After each replicate line had evolved for more than 1,200 generations, we isolated single clones from each population and determined their growth rates. All isolates grew faster than MEcoli_ref_1 (Extended Data Fig. 1). The best-performing isolate, which evolved for a total of about 1,240 generations (434 d), exhibited a doubling time of 4.3 ± 0.1 h (mean ± s.d.) and grew to an optical density that was 60% higher than MEcoli_ref_1 (Fig. 2b). This new reference strain, MEcoli_ref_2, isolated from line D, was used for all further experiments in this work.

### Adaptations of MEcoli_ref_2 for faster growth on methanol

We set out to gain an understanding of the genetic and physiological adaptations that improved the growth of MEcoli_ref_2 (Supplementary Data Table 2) on methanol compared with the initial methylotrophic population. Independent parallel evolutions of a defined ancestor frequently lead to the fixation of mutations in the same genetic targets and are responsible for fitness increases^50–52^. Thus, to identify mutations correlated with improved methylotrophic growth, we determined the metagenomic makeup of the evolving serial dilutions (D, 1,205; E, 1,130, F, 1,118; G, 1,119 generations; Fig. 2a) and identified repeatedly mutated loci. On average, each replicate line accumulated 316 ± 57 (mean ± s.d.) nonsynonymous and intergenic mutations compared with the initial methylotrophic population (Supplementary Data Table 3). While little evolutionary parallelism was observed at the nucleotide level, we found that the functional metabolic units methanol oxidation, RuMP cycle and pyruvate metabolism were repeatedly hit. This was also reflected at the gene and intergenic region level where we identified eight genes and intergenic loci that were independently mutated in all replicate lines (Fig. 2c,d and Supplementary Data Tables 3 and 4). In line with previous findings^50–52^, this suggests that mutations in these targets are drivers of improved growth on methanol.

To elucidate changes to central carbon metabolism, we examined mutated enzymes in vitro and integrated theoretical considerations, in silico modelling and previous insights.

**Extended Data Fig. 2:**
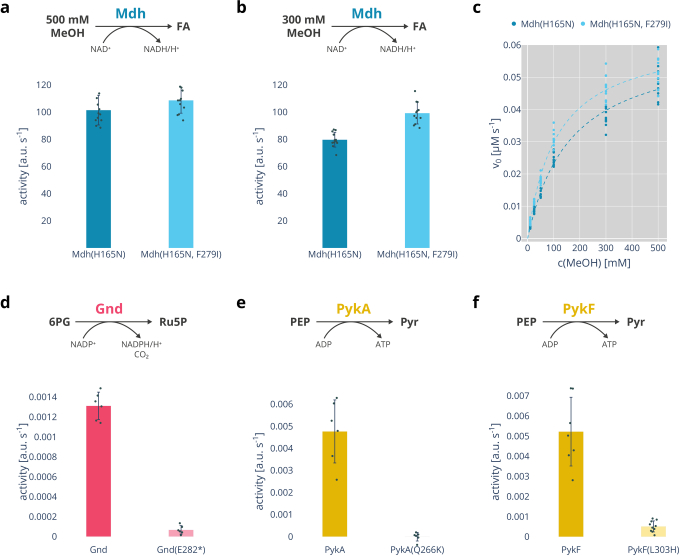
In vitro characterization of mutated metabolic proteins. All bars represent mean values of several technical replicates, error bars represent the associated standard deviation. All assays were conducted at 37 °C in a microplate reader. Initial reaction velocities (that is, enzyme activities) were determined by fitting lines through the initial constant increase or decrease of absorbance. **a** Activity of Mdh(H165N) and Mdh(H165N, F279I) at 500 mM methanol and 5 mM NAD^+^. At 500 mM methanol no significant difference between the two enzyme variants could be observed (two-sided Welch t-test *P* value = 0.1, n = 12 technical replicates). **b** Activity of Mdh(H165N) and Mdh(H165N, F279I) at 300 mM methanol and 5 mM NAD^+^. At 300 mM methanol Mdh(H165N, F279I) was 25 ± 13% faster than Mdh(H165N) (two-sided Welch t-test *P* value = 1.0 ⋅ 10^-6^, Cohen’s d effect size 0.38, n = 12 technical replicates). **c** Activities of Mdh(H165N) and Mdh(H165N, F279I) were determined at varying methanol concentrations to determine their kinetic parameters. Both enzymes exhibit Michaelis-Menten type kinetics: k_cat_(H165N) = 0.021 ± 0.001 s^-1^, K_M_(H165N) = 164 ± 13 mM, k_cat_(H165N, F279I) = 0.022 ± 0.001 s^-1^, K_M_(H165N, F279I) = 112 ± 6 mM (mean ± S.D.). For each methanol concentration n = 12 technical replicates were measured. **d** Gnd and Gnd(E282*) activity was measured at 5 mM 6-phosphogluconate and 1 mM NADP^+^. Gnd(E282*) exhibited 5.5 ± 3.3% (mean ± S.D.) activity of Gnd (two-sided Welch t-test *P* value = 8 ⋅ 10^-7^, Cohen’s d effect size 0.9, n = 6 technical replicates). **e** PykA and PykA(Q266K) activities were measured at 1 mM phosphoenolpyruvate and 1 mM ADP. PykA(Q266K) exhibited 0.0 ± 0.0% (mean ± S.D.) of PykA activity (two-sided Welch t-test *P* value = 4 ⋅ 10^-4^, Cohen’s d effect size 1.2, n = 6 technical replicates). **f** PykF and PykF(L303H) activities were measured at 1 mM phosphoenolpyruvate, 1 mM ADP and 1 mM fructose 1,6-bisphosphate. PykF(L303H) exhibited 10.0 ± 0.1% (mean ± S.D.) of PykF activity (two-sided Welch t-test *P* value = 3 ⋅ 10^-4^, Cohen’s d effect size 1.2, n = 12 technical replicates). Three very noisy PykF measurements, presumably due to air bubbles in the reaction mixtures, were excluded. Source data

Methanol oxidation was targeted by two genetic alterations conferring amino acid substitutions in methanol dehydrogenase and several intergenic mutations in the methanol dehydrogenase-encoding plasmid (i_*mdh*_; Fig. 2c). The latter probably increased methanol dehydrogenase expression^44^. Mutations in methanol dehydrogenase were present in three of the four replicate lines (F279I in lines D and E, and V359E in line G). Clonal isolates from line F, in which the mutations were not found, grew slower than the others on average (two-sided Welch *t*-test *P* value = 0.04, Cohen’s *d* effect size 1.0; Extended Data Fig. 1a). To further investigate the impact of the mutations in methanol dehydrogenase, we determined the kinetic parameters of the methanol dehydrogenase (Mdh) variant present in the initial methylotrophic population (Mdh(H165N)) and in MEcoli_ref_2 (Mdh(H165N, F279I)) towards methanol. The amino acid substitution did not alter the turnover number of the enzyme (*k*_cat_(H165N) = 0.021 ± 0.001 s^−1^, *k*_cat_(H165N, F279I) = 0.022 ± 0.001 s^−1^ (mean ± s.d.)), but reduced its Michaelis–Menten constant (K_M_(H165N) = 164 ± 13 mM, K_M_(H165N, F279I) = 112 ± 6 mM; Extended Data Fig. 2a–c). The higher methanol affinity increases the enzyme’s activity at methanol concentrations below 500 mM. For example, the enzyme activity of Mdh(H165N, F279I) at 300 mM was 25 ± 13% higher than that of Mdh(H165N). While the starting methanol concentration during serial dilution evolution was 500 mM, evaporation (methanol evaporation rate at 37 °C was ~2–3 mM h^−1^) and consumption reduced the available methanol over time, which selected for higher affinity towards methanol. Taken together, the data suggest that the mutations in methanol dehydrogenase confer faster growth on methanol under the chosen batch-growth conditions.

**Extended Data Fig. 3:**
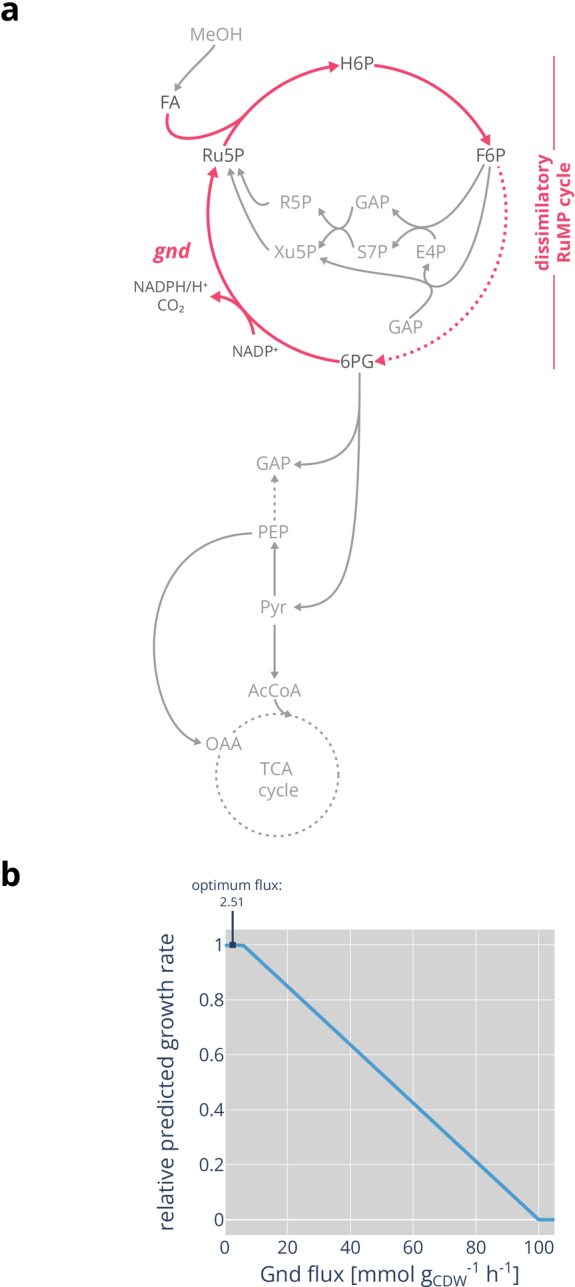
Dissimilatory RuMP cycle. **a** Schematic of the dissimilatory RuMP cycle. 6-phosphogluconate dehydrogenase (*gnd*) is essential for the dissimilatory RuMP cycle which dissipates formaldehyde, formed from methanol, to CO_2_ and NADPH. **b** pFBA-predicted growth of *E. coli* on methanol via the ED/TA RuMP cycle relative to flux through 6-phosphogluconate dehydrogenase. pFBA is a variant of regular flux balance analysis. First the algorithm determines the maximum possible growth rate given a set substrate uptake rate. Among all flux distributions that maximize growth, it then finds the set of fluxes that minimize the total flux through all reactions. Lower total flux is assumed to require less enzyme mass. Consequently, pFBA results reflect evolutionarily optimal solutions and have been shown to accurately predict fluxes in well-adapted organisms^57^. After having evolved for more than a thousand generations on methanol, we considered our methylotrophic *E. coli* well-adapted to its environment. Maximum possible flux is 100 mmol g_CDW_^-1^ h^-1^. For optimal growth small flux is predicted through 6-phosphogluconate dehydrogenase. Increasing 6-phosphogluconate dehydrogenase activity linearly decreases the predicted growth rate. In reality, we expect growth to cease earlier due to accumulation of NADPH and consequent redox imbalance which the model does not account for. Source data

Genetic elements of the RuMP cycle gained mutations in the promoter region of the *hps*-*phi* operon (p_*hps*-*phi*_) and in the gene encoding 6-phosphogluconate dehydrogenase (*gnd*). Alterations in the promoter of the *hps*-*phi* operon presumably fine-tuned expression of 3-hexulose 6-phosphate synthase and 6-phospho 3-hexuloisomerase, which are crucial for the efficient assimilation of formaldehyde into the RuMP cycle^44^. Maintaining low intracellular formaldehyde concentrations is required to prevent toxic protein–protein and protein–DNA crosslinking^45,53,54^ as well as to ensure efficient methanol oxidation in the thermodynamically disadvantaged methanol dehydrogenase reaction^19,55^. 6-Phosphogluconate dehydrogenase catalyses the decarboxylation of 6-phosphogluconate to ribulose 5-phosphate and is part of the so-called dissimilatory RuMP cycle, which oxidizes formaldehyde to CO_2_ instead of generating biomass precursors (Extended Data Fig. 3a)^56^. In natural methylotrophs, the pathway serves the generation of NADPH and formaldehyde detoxification^56^. We expected high flux through the 6-phosphogluconate dehydrogenase-catalysed reaction to be deleterious since it results in loss of carbon equivalents in the form of CO_2_ and reduces production of the RuMP cycle product pyruvate. To substantiate this notion, we modelled the *E. coli* methanol metabolism using parsimonious flux balance analysis (pFBA), a variant of conventional flux balance analysis that has been shown to accurately predict fluxes in *E. coli* subjected to long-term adaptive evolution (see Extended Data Fig. 3b for more details)^57^. For optimal growth, pFBA predicted little flux through the 6-phosphogluconate dehydrogenase reaction (that is, the reaction carries less than 10% of total flux away from the reaction’s substrate, 6-phosphogluconate; Fig. 2d and Supplementary Data Table 5). Eliminating the enzyme activity had little impact on the predicted growth rate because NADPH generation could be compensated by pyridine nucleotide transhydrogenase (~99% of optimum growth rate; Supplementary Data Table 6). In contrast, increasing flux through the 6-phosphogluconate dehydrogenase-catalysed reaction linearly decreased the predicted growth (Extended Data Fig. 3b). Two of the four observed mutations in *gnd* resulted in premature stop codons, and the other two are implicated in loss of function^58^. Indeed, the 6-phosphogluconate dehydrogenase (Gnd) variant in MEcoli_ref_2, that is, Gnd(E282*), exhibited strongly reduced activity towards 6-phosphogluconate in vitro (5.5 ± 3.3% (mean ± s.d.) of Gnd activity, two-sided Welch *t*-test *P* value = 8 × 10^−7^, Cohen’s *d* effect size 0.9; Extended Data Fig. 2d). The predicted sensitivity of growth to high Gnd activity together with the repeated fixation of loss-of-function mutations suggests that dysregulation of the reaction incurs a fitness cost.

Three mutations impacted the pyruvate node. We had noted earlier that carbon entry into the TCA cycle in MEcoli_ref_1 occurred via a carboxylating reaction^44^. This is consistent with our pFBA analysis that predicted carbon flow from pyruvate to phosphoenolpyruvate followed by its condensation with CO_2_ to oxaloacetate (Fig. 2d and Supplementary Data Table 5). Importantly, to avoid the creation of a futile cycle, the reverse reaction from phosphoenolpyruvate to pyruvate cannot carry flux (Fig. 2d). In line with this, we found that the genes encoding this reaction, *pykA* and *pykF*, acquired several independent nonsense and missense mutations in all replicate lines. In vitro assays of the mutant variants of either enzyme (pyruvate kinase 2 (PykA) or pyruvate kinase 1 (PykF)) found in MEcoli_ref_2 confirmed the loss of function. PykA(Q266K) exhibited no activity (0 ± 0% (mean ± s.d.) of PykA activity, two-sided Welch *t*-test *P* value = 4 × 10^−4^, Cohen’s *d* effect size 1.2; Extended Data Fig. 2e) and PykF(L303H) was severely impaired (10.0 ± 0.1% (mean ± s.d.) of PykF activity, two-sided Welch *t*-test *P* value = 3 × 10^−4^, Cohen’s *d* effect size 1.2; Extended Data Fig. 2f). Flux from pyruvate to phosphoenolpyruvate was probably further optimized by disruption of the phosphoenolpyruvate synthetase regulatory protein. The encoding gene, *ppsR*, was truncated or acquired mutations resulting in amino acid changes in all replicate lines and MEcoli_ref_2. Loss of phosphoenolpyruvate synthetase regulatory protein activity prevents the phosphorylation of phosphoenolpyruvate synthetase and, consequently, its deactivation^59^. Overall, evolution for more than 1,200 generations tuned the pyruvate metabolism to prevent adenosine triphosphate (ATP) loss through futile cycling.

Apart from genetic changes directly affecting methanol metabolism, two additional genes were mutated in all replicate lines, *rhlB* and *fepA*.

**Extended Data Fig. 4:**
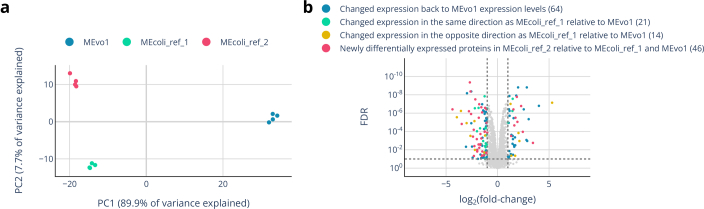
Proteomics of methylotrophic *E. coli* strains. **a** Principal component analysis of the protein abundances of the ancestral methanol-dependent *E. coli* strain, MEvo1, the original methylotrophic reference strain MEcoli_ref_1 (ref. ^44^) and the new, faster growing methylotrophic reference strain, MEcoli_ref_2. Plotted are the individual replicates of each strain. Each strain occupies a specific location in two-dimensional feature space indicating that the proteomes are quantitatively different to each other. Furthermore, the proteomes of the two methylotrophic *E. coli* strains, MEcoli_ref_1 and MEcoli_ref_2, are more similar to each other than either are to the non-methylotrophic ancestral strain. **b** Volcano plot of the proteome changes of MEcoli_ref_2 compared to MEcoli_ref_1. In total 145 proteins were differentially expressed at a log_2_(fold-change) cutoff of 1 and a false discovery rate (FDR) of 10^−1^. In MEcoli_ref_1 the expression patterns of many enzymes of central carbon metabolism changed compared to the ancestral methanol-dependent *E. coli* strain over the course of evolution^44^. For example, methanol dehydrogenase and enzymes of the RuMP cycle were strongly upregulated to facilitate methanol assimilation. Further evolution did not change expression of these enzymes in MEcoli_ref_2. Among the differentially expressed proteins in MEcoli_ref_2, 64 returned to levels found in the ancestral non-methylotrophic *E. coli* (MEvo1), 21 were previously differentially expressed in MEcoli_ref_1 and changed their expression in the same direction (for example, these are proteins that were both up/downregulated in MEcoli_ref_1 relative to MEvo1 and also up/downregulated in MEcoli_ref_2 relative to MEcoli_ref_1), 14 proteins inverted their expression pattern (that is, proteins that were overexpressed in MEcoli_ref_1 relative to MEvo1 and downregulated in MEcoli_ref_2 relative to MEcoli_ref_1 or vice versa) and 46 proteins were not already differentially expressed in MEcoli_ref_1 relative to MEvo1. Source data

The *rhlB* gene encoding an ATP-dependent RNA helicase was mutated or truncated, indicating a loss of function, in all serial dilution replicates and MEcoli_ref_2. The protein is a component of the *E. coli* degradosome and is involved in RNA^60^ and ribosome^61^ homeostasis as well as modulating the proteome composition^62^. Mutations in *rhlB* were fixed in all evolution lines after the isolation of MEcoli_ref_1 (Fig. 2c) and may have resulted in systemic proteome changes in MEcoli_ref_2. When we compared the protein abundances in the two MEcoli_ref strains, their proteomes exhibited distinct compositions (determined using principal component analysis; Extended Data Fig. 4a) and 145 differentially expressed proteins (Extended Data Fig. 4b). To identify cellular processes that adapted their expression patterns, we performed gene ontology (GO) gene set enrichment analysis. Among the 14 enriched GO terms, most revolved around ribosome biogenesis, whose associated proteins were consistently upregulated (Supplementary Data Table 7). In *E. coli*, the growth rate is proportional to the ribosome fraction^63^. However, the absence of carbon sources other than methanol triggers the stringent response in *E. coli* and results in the downregulation of ribosomes^64^. Consequently, ablating the degradosome activity by mutating *rhlB* may elevate ribosome biosynthesis and thus promote growth.

Amino acid changes in *fepA* encoding a ferric enterobactin outer membrane transporter may have increased iron uptake. It has previously been shown that iron can be growth-limiting in the minimal medium utilized in this study^65^.

### Production of value-added compounds from methanol

**Fig. 3:**
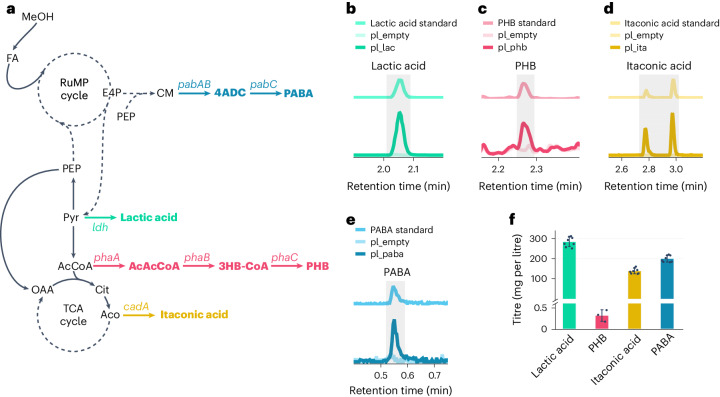
Bioconversion of methanol to products from four distinct metabolic nodes. **a**, Metabolic pathways for the production of PABA, L-lactic acid, PHB and itaconic acid. E4P and PEP are precursors to the biosynthesis of PABA via the chorismate (CM) pathway and the action of aminodeoxychorismate synthase (encoded by the gene *pabAB*) and aminodeoxychorismate lyase (encoded by the gene *pabC*). L-Lactate dehydrogenase (encoded by the gene *ldh*) catalyses the reaction from pyruvate to L-lactic acid. For PHB production from AcCoA, the *phaCAB* operon needs to be expressed. The gene *phaA* encodes an AcCoA acetyltransferase, which condenses two AcCoA molecules to acetoacetyl CoA (AcAcCoA). AcAcCoa is reduced by AcAcCoA reductase, encoded by *phaB*, to (*R*)-3-hydroxybutanoyl-CoA (3HB-CoA). Lastly, poly(3-hydroxyalkanoate) polymerase, encoded by *phaC*, generates PHB. Itaconic acid is synthesized by decarboxylating Aco via *cis*-aconitate decarboxylase (encoded by *cadA*). 4ADC, 4-amino-4-deoxychorismate. **b**–**e**, MEcoli_ref_2 transformed with pl_lac produced lactic acid (**b**), with pl_phb produced PHB (**c**), with pl_ita produced itaconic acid (**d**) and with pl_paba synthesized PABA (**e**). For each of the products shown, the traces of a representative sample, a commercial standard and the negative control are shown for comparison. In **c**, for analysis via gas chromatography using a flame ionization detector (GC-FID), PHB was depolymerized and derivatized to methyl 3-hydroxybutanoate. In **d**, the derivatization of itaconic acid generates two different isomers, which were detected here. In **b** and **d**, for detection and quantification, organic acids present in the medium supernatant were derivatized to their respective 3-nitrophenylhydrazones and measured using liquid chromatography coupled with mass spectrometry (LC/MS). All LC/MS chromatograms were selected for the theoretical values of the mass-to-charge ratio (*m*/*z*) of the respective compounds of interest. The gray shaded areas highlight the peak of each product of interest. **f**, Titres of the produced lactic acid, PHB, itaconic acid and PABA. Data are shown as the mean values ± s.d. for *n* = 8 (lactic acid, itaconic acid and PABA) or *n* = 3 (PHB) replicate inoculations of MEcoli_ref_2 transformed with the respective production plasmids. Source data

Following the generation of a fast-growing, synthetic methylotrophic *E. coli* strain, we aimed to investigate the potential of MEcoli_ref_2 for the bioconversion of methanol to value-added products. We selected four compounds as targets: lactic acid from pyruvate, PHB from AcCoA, itaconic acid from the TCA cycle and PABA from the chorismate pathway (Fig. 3a). Aromatics, such as PABA, serve as key intermediates in the chemical industry^42,66^, whereas the other three compounds are used to produce bioplastics that have large and growing markets^67–69^. Using our selection, we probed the achievable product spectrum of our synthetic methylotroph as pyruvate, AcCoA, the TCA cycle and the chorismate pathway constitute distinct metabolic nodes from which many biosynthesis pathways start. Furthermore, PABA is an interesting target because of its higher theoretical yield when produced from methanol (100% carbon yield) than from glucose (74.1%, all other compounds 100% theoretical carbon yield independently of carbon source; utilization of the ED/TA RuMP cycle does not negatively impact theoretical product yields compared with the fructose bisphosphate/aldolase variant, Supplementary Data Table 8).

**Extended Data Fig. 5:**
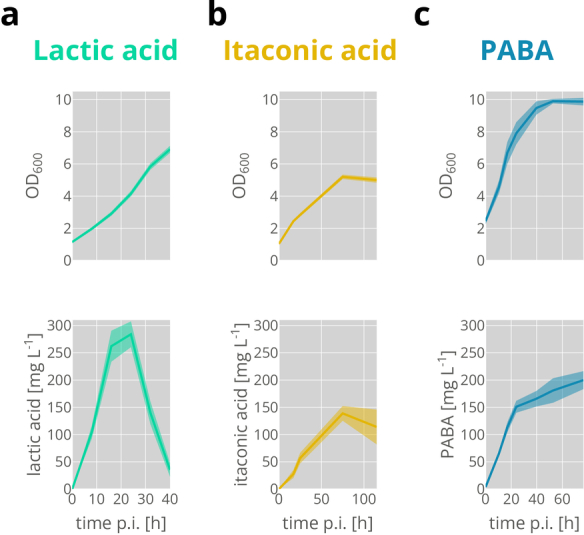
Time course production data. Shown are the means of n = 8 technical replicates and the standard deviation error bands around the means. **a** Shake flask lactic acid production (MEcoli_ref_2 + pl_lac). **b** Shake flask itaconic acid production (MEcoli_ref_2 + pl_ita). **c** Shake flask *p*-aminobenzoic acid production (MEcoli_ref_2 + pl_paba). **a-c** Time was measured post-induction (p.i.). Source data

The conversion of pyruvate to lactic acid requires only one enzyme, making it a promising first target for methanol-to-product transformation (Fig. 3a). For the bioproduction of lactic acid in *E. coli*, L-lactic acid dehydrogenase from *Streptococcus bovis* (*ldh*) has been used in the past^70,71^. We cloned the gene under control of an anhydrotetracycline-inducible (aTc-inducible) promoter into an expression plasmid (pl_lac; Supplementary Data 1) and transformed it into MEcoli_ref_2. Growth and induction of the *ldh*-expressing strain in minimal medium supplemented with 500 mM methanol resulted in a maximum titre of 284.0 ± 23.6 mg l^−1^ (3.2 ± 0.3 mM) for lactic acid with a carbon yield of 15.7 ± 1.0% (0.147 ± 0.009 g_lactic acid_ g_MeOH_^−1^) and a productivity of 12.7 ± 1.0 mg l^−1^ h^−1^ (mean ± s.d.; Fig. 3b,f and Extended Data Fig. 5a) after 22 h. Quantifiable amounts of lactic acid were not detected in the empty plasmid negative control (pl_empty; Supplementary Data 2). Prolonged incubation resulted in a subsequent decrease in lactic acid concentration in the medium (Extended Data Fig. 5a), presumably due to the activity of native *E. coli* L-lactate dehydrogenase (*lldD*)^72^. Methanol was the only available carbon source in the medium used for cultivation of MEcoli_ref_2, indicating that lactic acid was produced from methanol. Nevertheless, to exclude the possibility that unaccounted carbon sources contributed to the formation of lactic acid, we grew L-lactate dehydrogenase-expressing MEcoli_ref_2 under the same conditions in minimal medium containing 500 mM ^13^C methanol (99% atomic purity). The produced lactic acid matched the expected 97% labelling (Supplementary Data Table 9).

PHB is produced from AcCoA and requires the heterologous expression of three genes in *E. coli* (Fig. 3a). We sourced the required genes (*phaCAB*) from the native PHB producer *Cupriavidus necator* H16 and cloned them into the above-described expression vector (pl_phb; Supplementary Data 3). Following the transformation of pl_phb into MEcoli_ref_2, expression of the *phaCAB* operon resulted in the production of approximately 0.32 ± 0.11 mg l^−1^ (mean ± s.d.) PHB after 74 h (Fig. 3c,f).

The synthesis of itaconic acid proceeds from the TCA cycle intermediate *cis*-aconitate (Fig. 3a). The reaction is catalysed by *cis*-aconitate decarboxylase. To enable production, we cloned the gene encoding the enzyme (*cadA*) from the industrial itaconic acid producer *Aspergillus terreus*^69^ into the same vector as before (pl_ita; Supplementary Data 4). Subsequently, MEcoli_ref_2 carrying pl_ita produced up to 138.9 ± 13.5 mg l^−1^ (1.1 ± 0.1 mM) itaconic acid after 75 h with a carbon yield of 6.0 ± 0.7% (0.048 ± 0.006 g_itaconic acid_ g_MeOH_^−1^) and a productivity of 1.85 ± 0.18 mg l^−1^ h^−1^ (mean ± s.d.; Fig. 3d,f and Extended Data Fig. 5b).

Lastly, we attempted the conversion of methanol to PABA. PABA is produced from erythrose 4-phosphate and phosphoenolpyruvate via the chorismate pathway and the action of aminodeoxychorismate synthase (encoded by *pabAB*) and aminodeoxychorismate lyase (encoded by *pabC*; Fig. 3a). Its biosynthesis draws on two metabolic nodes, lower glycolysis and the RuMP cycle. Importantly, production requires anaplerotic replenishment of RuMP cycle intermediates to prevent this essential metabolic system from stalling. To test if methanol metabolism in MEcoli_ref_2 can overcome this metabolic challenge and support the production of aromatics, we introduced the requisite genes into MEcoli_ref_2 (pl_paba encoding *pabAB* from *Corynebacterium efficiens* YS-314 and *pabC* from *E. coli*; Supplementary Data 5) and assayed for the biosynthesis of PABA. The resulting strain produced 199.9 ± 16.4 mg l^−1^ (1.5 ± 0.1 mM) PABA after 77 h with a carbon yield of 3.0 ± 0.0% (0.018 ± 0.00 g_PABA_ g_MeOH_^−1^) and a productivity of 2.56 ± 0.23 mg l^−1^ h^−1^ (mean ± s.d.; Fig. 3e,f and Extended Data Fig. 5c). In summary, we show the production of targeted chemicals from methanol using the synthetic methylotrophic *E. coli* strain MEcoli_ref_2.

### Improved production through high-cell-density cultivation

**Fig. 4:**
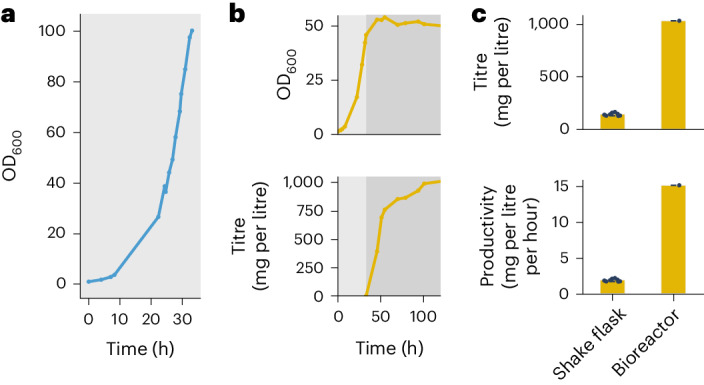
Improved itaconic acid production through high-cell-density cultivation. Bioreactor growth for high-cell-density and precision fermentation production was assayed in a 3.6 litre bioreactor containing minimal medium (1.4 litres). Methanol was fed to maintain concentrations between 500 and 1,000 mM and to compensate for consumption and evaporation. **a**, MEcoli_ref_2 growth to an industrially relevant cell density. MEcoli_ref_2 achieved an OD_600_ of 100.2 (22.1 ± 0.5 g_CDW_ l^−1^ (mean ± s.d.)) on methanol under fed-batch conditions while maintaining a high growth rate (*T*_d_ = 4.9 h). **b**, Bioreactor production of itaconic acid. MEcoli_ref_2 transformed with pl_ita was grown to an OD_600_ of 46 (light grey shaded area). Subsequently, itaconic acid production was induced and monitored over time (dark grey shaded area). **c**, Itaconic acid production in the bioreactor compared with shake flasks. In the bioreactor, the titre (1.0 g l^−1^) and productivity (15.0 mg l^−1^ h^−1^) were around sevenfold (7.4 ± 0.7) and eightfold (8.1 ± 0.8) higher than in the shake flasks, respectively. Data are shown as the mean values ± s.d. for *n* = 8 replicate inoculations of MEcoli_ref_2 transformed with pl_ita for shake-flask cultivation and for one replicate of the bioreactor experiment. Source data

Economical industrial production processes require high cell densities to achieve high space–time yields. To demonstrate that synthetic methylotrophy in *E. coli* is, in principle, suited for precision fermentation, we grew MEcoli_ref_2 in a bioreactor under fed-batch conditions. Following standard bioreactor protocols, MEcoli_ref_2 grew exponentially to an OD_600_ of 100.2 (22.1 ± 0.5 g_CDW_ l^−1^ (mean ± s.d.; CDW, cell dry weight)) at a doubling time of 4.9 h (Fig. 4a). At the achieved biomass concentration, oxygen transfer became limiting. Next, we probed if higher biomass translates to an enhanced performance using the itaconic acid-producing strain. We grew MEcoli_ref_2 transformed with pl_ita to an OD_600_ of 46 and subsequently induced enzyme expression resulting in around a sevenfold higher itaconic acid titre of 1.0 g l^−1^ (7.7 mM) and an eightfold increased productivity of 15.0 mg l^−1^ h^−1^ compared with shake flasks (Fig. 4b,c). Importantly, the performance parameters increased proportionally with the biomass concentration, suggesting that shake-flask production can be efficiently scaled up. Overall, we can demonstrate the capability of MEcoli_ref_2 to grow and produce value-added products under precision fermentation conditions.

## Discussion

Synthetic methylotrophy in model organisms offers an opportunity for the valorization of greenhouse gases to value-added chemicals. Previous work from our laboratory in which we generated an *E. coli* strain that utilizes the RuMP cycle for growth on methanol as the sole carbon and energy source^44^ was a promising starting point to advance the biotechnological application of synthetic methylotrophs towards carbon-negative chemicals.

In this work, we generated a methylotrophic *E. coli* strain, MEcoli_ref_2, that grows at a doubling time of 4.3 ± 0.1 h (mean ± s.d.). This growth rate compares favourably with other synthetic methylotrophic model organisms^27,43,45,73,74^ and is 46% faster than our originally reported reference strain MEcoli_ref_1 (ref. ^44^). Notably, the doubling time of MEcoli_ref_2 is lower than that of the natural model methylotroph *B. methanolicus* at 37 °C (*T*_d_ = 5 h (ref. ^48^)), which also utilizes an NAD-dependent methanol dehydrogenase and the RuMP cycle^75^. Furthermore, the growth of MEcoli_ref_2 is independent of costly medium supplements such as vitamin B_12_ and biotin, which are required for *B. methanolicus* cultivation^76^. Faster growth on methanol via the RuMP cycle is possible, although it generally requires higher temperatures (for example, *B. methanolicus* at 50 °C has a doubling time of 1.7 h (ref. ^48^)) or methanol oxidation via PQQ-dependent (pyrroloquinoline quinone-dependent) methanol dehydrogenase that has superior kinetics and a higher thermodynamic driving force^47,55,77^ (for example, *Methylobacillus flagellatus* at 37 °C has a doubling time of ~1 h (ref. ^78^)). Utilization of the latter, however, reduces the theoretical biomass yield^46^.

The MEcoli_ref_2 strain was generated through the long-term adaptive evolution of the original methylotrophic population (*T*_d_ ≈ 60 h (ref. ^44^)) under a serial dilution regime. It was isolated after about 1,240 generations (434 d; Fig. 2a) from one of four replicate lines (line D). Evolution was highly parallel and pointed at nine genetic targets driving adaptation towards accelerated methanol assimilation (Fig. 2c and Supplementary Data Tables 3 and 4). The methanol oxidation capacity increased through higher methanol dehydrogenase expression (i_*mdh*_)^44^ and improved kinetics (Mdh(H165N, F279I)). The fine-tuned expression of RuMP cycle enzymes (p_*hps*-*phi*_), the disruption of futile cycles (*gnd*, *pykA*, *pykF*) and, presumably, the boosted activity of phosphoenolpyruvate synthetase (via *ppsR*) rendered methanol assimilation more efficient (Fig. 2 and Extended Data Figs. 2 and 3). To accommodate the improved metabolic potential for faster growth on methanol, the disruption of degradosome-associated ATP-dependent RNA helicase RhlB may have released dysregulated ribosome biogenesis. Future reconstruction work will be necessary to determine the exact genetic makeup required for the growth of *E. coli* on methanol and the factors that enhance the speed of methanol assimilation.

Green methanol bioconversion to chemicals is a promising approach to decarbonize chemical production^47^. Building on MEcoli_ref_2, we can show the proof-of-concept production of lactic acid, PHB, itaconic acid and PABA from methanol (Fig. 3f) with carbon yields for the soluble products ranging from 3.0 to 15.7%. Notably, MEcoli_ref_2 supported the production of PABA despite it depleting RuMP cycle intermediates. The successful biosynthesis of all four compounds demonstrates the robustness of methanol metabolism in MEcoli_ref_2, and indicates its potential to produce a range of different products. Furthermore, it fulfils one of the original promises of synthetic methylotrophy in model organisms—the ‘plug and play’ of existing production pathways.

Economical production processes require high cell densities to achieve sufficient performance. MEcoli_ref_2 was capable of growing to an industrially relevant OD_600_ of 100 on methanol using a regular bioreactor at a growth rate that is comparable to shake flasks. Higher oxygen transfer rates would probably have improved the growth further. Importantly, high biomass concentrations translated to increased titres and productivities. With the observed behaviour of MEcoli_ref_2 under bioreactor conditions, we conclude that the synthetic methylotrophic *E. coli* strain is amenable to precision fermentation processes.

The synthetic methylotrophic *E. coli* introduced here is in the early stages of development towards commercial production, but the achieved titres, yields and productivities overall compare favourably with other biosynthesis platforms at similar technological readiness levels. The lactic acid titre (284.0 ± 23.6 mg l^−1^) and the carbon yield (15.7 ± 1.0%) were higher than achieved using a synthetic formatotroph via the reductive glycine pathway (108 mg l^−1^, ~6% carbon yield)^73^. Expending a greater engineering effort, a higher titre (3.5 g l^−1^) from methanol at a similar yield (23.5%) was achieved in *Pichia pastoris*^79^, which together with *Ogataea polymorpha* has been explored as a host for methanol bioproduction in recent years^80–83^. Itaconic acid biosynthesis has previously been reported in the natural methylotroph *Methylorubrum extorquens*, although the titre was lower (31.6 mg l^−1^ (ref. ^84^)) than demonstrated here. Our achieved performance parameters for itaconic acid and PABA are similar to initial efforts for production from sugars^85,86^. Lastly, for carbon storage, PHB is naturally synthesized by certain methylotrophs that generally produce higher titres^87–89^. For economically viable processes, titres, yields and productivities will have to be increased substantially. Typically, titres in the region of hundreds of grams per litre, yields within 10–20% of the theoretical maximum and productivities in the range of grams per litre per hour are required. Nevertheless, many such optimization campaigns have previously been undertaken in *E. coli*, which will de-risk and speed up future development.

Overall, we generated a synthetic methylotrophic reference strain, MEcoli_ref_2, and achieved growth on par with the natural methylotroph *B. methanolicus*. To establish bioproduction from methanol, we engineered MEcoli_ref_2 for the production of commodity chemicals from four key biosynthetic starting points. Future work will enable economical bioprocesses by optimizing the production pathways and improving high-cell-density cultivation in bioreactors. Ultimately, our work lays the foundation for the efficient valorization of green methanol and, thus, greenhouse gases.

## Methods

### Chemicals and reagents

A list of all reagents and commercial kits used for this study is provided in Supplementary Data Table 10.

M9 minimal medium used for the cultivation of methylotrophic *E. coli* contained the following salts (in grams per litre): Na_2_HPO_4_ (6.78), KH_2_PO_4_ (3.0), NaCl (0.5), NH_4_Cl (1.0), CaCl_2_ (0.011), MgSO_4_·7H_2_O (0.493) and trace elements. Trace elements were present in the medium at the following concentrations (in milligrams per litre): Na_2_EDTA (5.0), MnCl_2_ (4.2), FeSO_4_·7H_2_O (1.0), Co(NO_3_)_2_·6H_2_O (1.0), ZnSO_4_·7H_2_O (1.0), CuSO_4_·5H_2_O (0.1), Na_2_MoO_4_·2H_2_O (0.1) and NiCl_2_·6H_2_O (0.2). If indicated, antibiotics were added in the following concentrations (in micrograms per millilitre): ampicillin sodium salt (100), carbenicillin disodium salt (50), streptomycin sulfate (20), kanamycin monosulfate (50) and chloramphenicol (4.25, for *E. coli* DH5α: 34).

### Primers, plasmids and gene blocks

Supplementary Data Table 10 lists the primers, plasmids and gene blocks used in this study. Primers were synthesized by Microsynth. Polymerase chain reaction (PCR) processes were carried out using Q5 polymerase (New England Biolabs (NEB)) or Taq polymerase (Thermo Fisher Scientific) for cloning and confirmatory reactions, respectively. Gibson assemblies for cloning were performed using NEBuilder 2x HiFi DNA Assembly Master Mix (NEB), and KLD ligations with the NEB KLD Enzyme Mix. All synthetic gene blocks used in this study were synthesized by Twist Bioscience, and codon-optimized by their algorithm for expression in *E. coli*. Plasmids were propagated in *E. coli* DH5α (NEB) cultivated in lysogeny broth (LB medium) supplemented with appropriate selective antibiotics. The DNA sequence encoding the *phaCAB* operon was obtained via PCR from pHB-4 (Zhou Lab, plasmid number 140957, Addgene).

### Serial dilution evolution for improved methylotrophic growth

The early phase of the serial dilution experiment was as described previously^44^. Initially, the methylotrophic population from the chemostat was passaged seven times in a 20 ml volume of M9 minimal medium supplemented with 500 mM methanol, 0.1 mM isopropyl β-D-1-thiogalactopyranoside (IPTG), ampicillin and streptomycin. Subsequently, this culture was split into four replicate lines (D, E, F and G; Fig. 2a). Each line was propagated at 37 °C in a 30 ml sample of minimal medium containing 500 mM methanol in 100 ml baffled shake flasks and passaged into fresh medium once it had reached the late exponential or early stationary phase. In the beginning, cultures were incubated using a Minitron shaker (Infors-HT) at 160 revolutions per min (r.p.m.), 50 mm shaking throw. Later, they were moved to a Multitron shaker (Infors-HT) at 220 r.p.m., 25 mm throw. The antibiotics ampicillin and streptomycin were added to the medium in the beginning of the evolution experiment but were later omitted. Subsequently, antibiotics were occasionally added to test for contaminations. Detailed information on every replicate line, medium composition for every passage, and so on, are listed in Supplementary Data Table 1.

### Isolation and growth characterization of MEcoli_ref_2

Aliquots from each evolution replicate line were streaked out on minimal solid medium (M9 medium with 1.5% (w/v) agar) after 1,241 (line D), 1,236 (E), 1,218 (F) and 1,230 (G) generations. The Parafilm-sealed agar plates were incubated at 37 °C until colonies were clearly visible. Subsequently, six (lines D, E and G) or five (line F) colonies were inoculated in a 3 ml volume of minimal medium supplemented with 500 mM methanol. In addition, MEcoli_ref_1 was inoculated in the same medium from a glycerol cryostock. The cultures were incubated in a 24-deep-well plate sealed with Breathe-Easy membrane (Diversified Biotech) under conditions of 37 °C, 220 r.p.m. and 25 mm throw using a Multitron shaker until the exponential or early stationary phase. Next, the cultures were diluted to OD_600_ 0.01 in fresh medium and split into three aliquots of 150 μl in a 96-well plate. Each well was covered with 50 μl mineral oil to prevent evaporation^43^. The 96-well plate was membrane-sealed. Growth of the *E. coli* cultures was monitored by measuring the absorbance at 600 nm using a microplate reader at 800 r.p.m. (LogPhase 600, Agilent). Growth rates were determined using the Dashing Growth Curves application^90^. Growth curves were smoothed with a rolling window of 30 and the exponential growth phase was determined manually.

### Genome resequencing

Genome resequencing of population and clonal samples was described previously^44^. The protocol is reproduced here. For genome resequencing, about 2 OD units of cells were sampled, centrifuged for 1 min at 11,000 g and the supernatant discarded. Genomic DNA was extracted using a MasterPure DNA purification kit (Epicenter). Purified genomic DNA was sent for Illumina NovaSeq sequencing (BMKGene). The BBMap (v.38.95) clumpify function was used to filter raw reads for optical and PCR duplicates. The maximum distance to consider for optical replicates was set to the appropriate value for the used sequencer (dupedist = 12,000) and we allowed for one base substitution between duplicates (subs = 1). For PCR duplicates, the same substitution setting was used with two passes for error correction (passes = 2). Filtered reads were aligned to the reference genome of *E. coli* BW25113 (GenBank accession: CP009273) and the plasmid maps of pSEVA424 with *mdh2* CT4-1 from *C. necator* and pSEVA131 with *hps* and *phi* from *M. flagellatus* by Breseq (0.36.0)^91^ in clonal or population mode with default values for all other settings. Genome resequencing samples are summarized in Supplementary Data Table 11.

### Identification of repeatedly occurring mutations

Mutations in the population samples from the evolving serial dilution replicate lines were filtered for nonsynonymous and intergenic mutations. Only mutations present at greater than 60% frequency in the respective population were considered. Mutations already present in the original methylotrophic culture, the starter culture of the serial dilution evolution experiment, were discarded (Supplementary Data Table 12). The resulting list of genetic changes was grouped at the gene and intergenic region level (Supplementary Data Table 3). Similarly, nonsynonymous and intergenic mutations that are present in MEcoli_ref_2 but not MEcoli_ref_1 (Supplementary Data Table 13 (ref. ^44^)) were filtered and grouped.

### Enzyme expression and purification

The coding sequences of Mdh(H165N), Mdh(H165N, F279I), Gnd, Gnd(E282*), PykA, PykA(Q266K), PykF and PykF(L303H) were cloned into a pET16b expression vector using Gibson assembly (plasmid vector maps in Supplementary Data 6–13) such that ten His residues were added to the N terminus for nickel-immobilized metal affinity chromatography purification.

Protein expression and purification were performed as described previously^44^. The protocol is reproduced here. All proteins were treated identically unless stated otherwise.

For protein expression, pre-cultures were inoculated in a 10 ml sample of LB medium supplemented with carbenicillin and incubated at 37 °C, 220 r.p.m., 25 mm throw and diluted 1:50 (v/v) in 200 ml of the same medium in a one litre baffled shake flask on the following day. The cultures were grown to mid-exponential phase (OD_600_ ≈ 0.7) at 37 °C, 220 r.p.m., 25 mm throw. Once an OD_600_ of ~0.7 was reached, the cultures were induced with 0.3 mM IPTG and incubated overnight at 16 °C, 220 r.p.m., 25 mm throw. Cells were harvested via centrifugation (3,250 *g*, 30 min, 4 °C), resuspended in lysis buffer (15 ml; 50 mM NaH_2_PO_4_, 300 mM NaCl, 20 mM imidazole, 2 mM dithiothreitol, EDTA-free protease inhibitor cocktail (Roche cOmplete, Sigma-Aldrich)) and lysed via sonication (6 mm sonication probe, amplitude 30, process time 4 min, impulse time 5 s, cool-down time 25 s, (Q700 sonicator, Qsonica)). Cell debris were cleared via centrifugation (20,000 *g*, 40 min, 4 °C). His-tagged proteins were isolated from the resulting solutions using fast protein liquid chromatography (ÄKTA, HisTrap HP, GE Healthcare) using a linear gradient from starting buffer (lysis buffer without protease inhibitor cocktail) to elution buffer (starting buffer with 500 mM imidazole) over 20 min at a flow rate of 1 ml min^−1^. To stabilize PykA, 10 mM KCl was added to all buffers during purification^92^. Protein purity was confirmed using sodium dodecyl sulfate–polyacrylamide gel electrophoresis.

Buffer was exchanged to the reaction buffer (for Mdh: 100 mM 3-(*N*-morpholino)propanesulfonic acid (MOPS), 5 mM MgSO_4_, 10% (v/v) glycerol, pH 7.4; and for Gnd and PykA/F: 50 mM 4-(2-hydroxyethyl)-1-piperazineethanesulfonic acid (HEPES), 10 mM MgCl_2_, 10 mM KCl, pH 7.5) by repeated concentration in centrifugal filter units (Amicon Ultra, 10 kDA molecular mass cutoff, Sigma-Aldrich) and subsequent dilution in the reaction buffer until a total dilution factor of greater than 100,000 was achieved. The enzyme concentration was determined via absorbance measurements at 280 nm using a NanoDrop spectrophotometer.

### In vitro enzyme assays

All assays were performed in 96-well plate format using a microplate reader (BioTek Synergy H1, Agilent Technologies).

The methanol dehydrogenase activity was assayed in reaction buffer supplemented with 0.125 mg ml^−1^ Mdh(H165N)/Mdh(H165N, F279I), 5 mM NAD^+^ and 500 mM methanol at 37 °C. Assays were started by adding preheated methanol reaction buffer solution (10 μl) to preheated reaction buffer supplemented with enzyme and NAD^+^. The reaction was monitored by measuring the increase in NADH at 340 nm.

The 6-phosphogluconate dehydrogenase activity was assayed in reaction buffer supplemented with 5 μM Gnd/Gnd(E282*), 1 mM NADP^+^, 5 mM 6-phosphogluconate at 37 °C. Assays were started by adding preheated 6-phosphogluconate reaction buffer solution (20 μl) to preheated reaction buffer supplemented with enzyme and NADP^+^. The reaction was monitored by measuring the increase in NADPH at 340 nm.

The respective pyruvate kinase 1 or 2 activity was assayed in reaction buffer supplemented with 5.0 μg ml^−1^ PykF/PykF(L303H) or 4.4 μg ml^−1^ PykA/PykA(Q266K), 1 mM adenosine diphosphate, 1 mM phosphoenolpyruvate, 0.5 mM NADH, 3 U ml^−1^ L-lactate dehydrogenase (recombinant from *E. coli*, Sigma-Aldrich). The pyruvate kinase reaction was coupled to the lactate dehydrogenase activity, which converts pyruvate to lactate while consuming NADH. The reaction was monitored by measuring the decrease in NADH at 340 nm.

### Parsimonious flux balance analysis of methylotrophic growth

Parsimonious flux balance analysis was conducted on the *E. coli* core genome model, which contains 72 metabolites and 95 reactions^93^, using the COBRApy framework (v.0.29.0)^94^ under Python (v.3.12.0). To represent the methanol metabolism of the methylotrophic reference strain, the RuMP cycle genes *mdh*, *hps* and *phi* were added to the model. Genes *frmA* and *fdh* encode S-(hydroxymethyl)glutathione dehydrogenase and formate dehydrogenase, respectively. The sedoheptulose 1,7-bisphosphate variant of the RuMP cycle was also enabled^56^. The specific reactions are listed in Supplementary Data Table 14. MEcoli_ref_2 carries a deletion of *tpiA*, encoding triosephosphate isomerase. This deletion was also made in the core metabolic model. The pathways that convert pyruvate to malate and are catalysed by NAD or NADP-dependent malic enzyme were not considered in our *E. coli* metabolic model because neither support substantial flux in the carboxylating direction under physiological conditions and ambient carbon dioxide concentrations^95^.

### Proteomics

The ancestral methanol-dependent *E. coli* (MEvo1), MEcoli_ref_1 and MEcoli_ref_2 were grown as described previously^44^. The protocol is reproduced here. Each strain was streaked out on an agar plate containing minimal medium supplemented with 500 mM methanol and incubated at 37 °C until colonies were clearly visible. A cross-section of the colonies was used to inoculate a pre-culture in a 30 ml volume of minimal medium supplemented with 500 mM methanol and cultivated in baffled shake flasks at 37 °C, 220 r.p.m., 25 mm throw until the stationary phase. Next, the cultures were diluted 1:200 (v/v) into fresh medium, grown until the mid-exponential phase and split 1:100 (v/v) (MEvo1), 1:200 (MEcoli_ref_1) and 1:110 (MEcoli_ref_2) into four main-culture replicates. Once the cultures had reached the mid-exponential phase (OD_600_ ≈ 0.6 for MEcoli_ref_1 and MEcoli_ref_2, and OD_600_ ≈ 0.4 for MEvo1), 4 OD units (where 1 OD unit is equal to 1 ml culture at an OD_600_ of 1) of the cells were harvested, cooled to 4 °C, spun down (3,220 *g*, 15 min) and washed once with 10 mM MgCl_2_ (20 ml) and then with 10 mM MgCl_2_ (1 ml). Finally, the supernatant was discarded and the cell pellet was shock frozen in liquid nitrogen. The procedure was adapted for the ancestral strain to include 20 mM pyruvate, carbenicillin, streptomycin and 0.1 mM IPTG in all media.

To each cell pellet a 100 µl aliquot of lysis buffer (4% sodium dodecyl sulfate in 100 mM Tris/HCl pH 8.2) was added. Protein extraction was carried out using a tissue homogenizer (TissueLyser II, QUIAGEN) by applying two 2 min cycles at 30 Hz. The samples were treated with high intensity focused ultrasound for 1 min before centrifugation at 20,000 *g* for 10 min. The protein concentration was determined using the Lunatic ultraviolet/visible polychromatic spectrophotometer (Unchained Labs) with a 1:25 (v/v) dilution of each sample. For each sample protein (50 µg) was taken and reduced with 5 mM tris(2-carboxyethyl)phosphine and alkylated with 15 mM chloroacetamide at 30 °C for 30 min. Samples were processed using single‐pot solid‐phase-enhanced sample preparation (or SP3). SP3 protein purification, digest and peptide clean up was performed using a KingFisher Flex System (Thermo Fisher Scientific) and carboxylate-modified magnetic particles (GE Life Sciences; GE65152105050250, GE45152105050250)^96,97^. Beads were conditioned following the manufacturer’s instructions, which consisted of three washes with water at a concentration of 1 µg µl^−1^. Samples were diluted with 100% (v/v) ethanol to a final concentration of 60% (v/v) ethanol. The beads, wash solutions and samples were loaded into 96-deep-well or -micro-well plates and transferred to the KingFisher system. The following steps were carried out on the robot: collection of beads from the last wash, protein binding to beads, washing of beads in wash solutions 1–3 (80% (v/v) ethanol), protein digestion (overnight at 37 °C with a trypsin:protein ratio of 1:50 in 50 mM triethylammonium bicarbonate) and peptide elution from the magnetic beads using ultrapure water. The digest solution and water elution were combined and dried to completeness and re-solubilized in 20 µl of MS-grade sample buffer (3% acetonitrile, 0.1% formic acid). The peptide concentration was determined using the Lunatic ultraviolet/visible polychromatic spectrophotometer.

LC/MS/MS analysis was performed using an Orbitrap Fusion Lumos spectrometer (Thermo Scientific) equipped with a Digital PicoView ion source (New Objective) and coupled to an M-Class ultra-high-pressure liquid chromatography (UPLC) system (Waters). The solvent composition of the two channels was 0.1% (v/v) formic acid for channel A and 99.9% acetonitrile in 0.1% (v/v) formic acid for channel B. The column temperature was 50 °C. For each sample the equivalent of 0.3 absorbance was loaded on a commercial ACQUITY UPLC M-Class Symmetry C18 Trap Column (100 Å, 5 µm, 180 µm × 20 mm; Waters) connected to an ACQUITY UPLC M-Class HSS T3 Column (100 Å, 1.8 µm, 75 µm × 250 mm; Waters). The peptides were eluted at a flow rate of 300 nl min^−1^. After a 3 min initial hold at 5% (v/v) B, a gradient from 5 to 22% B in 80 min and from 22 to 32% B in an additional 10 min was applied. The column was cleaned after the run by increasing to 95% B and holding at 95% B for 10 min before re-establishing the loading condition. Samples were measured in a randomized order. For the analysis of the individual samples, the mass spectrometer was operated in data-independent acquisition (DIA) mode. DIA scans covered a range from 392 to 1,008 *m*/*z* in windows of 16 *m*/*z*. The resolution of the DIA windows was set to 30,000, with an automatic gain control target value of 500,000, the maximum injection time set to 50 ms and a fixed normalized collision energy of 33%. Each instrument cycle was completed by a full MS scan monitoring 350–1,500 *m*/*z* at a resolution of 120,000.

The MS proteomics data were handled using the local laboratory information management system B-Fabric^98^.

The acquired shotgun MS data were processed for identification and quantification using the DIA-NN software suite^99^. Spectra were searched using the database in FASTA file format consisting of the *E. coli* proteome, mutant proteins and common protein contaminants. Tandem mass tag modification on peptide N termini and lysine side chains and carbamidomethylation of cysteine were fixed modifications, while methionine oxidation was variable. Enzyme specificity was set to trypsin/P, enabling a minimal peptide length of seven amino acids and a maximum of two missed cleavages.

The package prolfqua^100^ was used to analyse the differential expression and to determine group differences, confidence intervals and false discovery rates for all quantifiable proteins. Starting with the report.tsv file generated by DIA-NN, which does report the precursor ion abundances for each raw file, we determined the protein abundances by first aggregating the precursor abundances to peptidoform abundances. Then, we used Tukey’s median polish to estimate protein abundances. Furthermore, before fitting the linear models, we transformed the protein abundances using variance stabilizing normalization^101^.

### Proteomics gene set enrichment analysis of GO terms

The differential protein expression data of MEcoli_ref_2 relative to MEcoli_ref_1 was analysed for GO term enrichment using clusterProfiler (4.1.0, R version 4.3.2).

### Theoretical yield calculations

The above-described metabolic model was utilized to compute the theoretical yields of lactic acid, PHB, itaconic acid and PABA by introducing the respective biosynthesis pathways and changing the objective function to the product of interest. Theoretical yields were determined for biosynthesis from glucose using wild-type *E. coli* metabolism and methanol using the ED/TA or the fructose bisphosphate aldolase/TA RuMP cycle.

### Plasmid transformation into MEcoli_ref_2

Transformation of vectors for bioproduction into methylotrophic *E. coli* was performed according to an adapted chemical transformation (heat shock) protocol^102,103^. A cryogenic glycerol stock of MEcoli_ref_2 was inoculated in M9 medium containing 500 mM methanol and incubated at 37 °C, 220 r.p.m., 25 mm throw until the exponential phase. Subsequently, it was diluted into a 30 ml volume of LB medium supplemented with streptomycin and ampicillin for maintenance of the methylotrophy plasmids and incubated overnight. Once OD_600_ was between 0.4 and 0.9, the cells were harvested via centrifugation at 3,200 *g*, 4 °C, 2 min, washed once with 30 mM calcium chloride (2 ml) and resuspended in 30 mM calcium chloride solution (200 µl). Plasmid (100 ng) was added to the cell suspension (50 µl) and incubated on ice for 30 min. Transformation was carried out via heat shock at 42 °C for 45 s, followed by incubation on ice for 2 min. The cells were subsequently incubated for 8–24 h at 37 °C, followed by 20-fold dilution into LB medium supplemented with streptomycin, ampicillin and chloramphenicol for selection of cells containing both the methylotrophy plasmids and the bioproduction plasmid. After a minimum increase in the optical density by a factor of four, cryogenic glycerol stocks of the transformants were created from the liquid cultures.

### Lactic acid, itaconic acid and PABA production

The cryogenic glycerol stocks of MEcoli_ref_2 + pl_lac (lactic acid production), MEcoli_ref_2 + pl_ita (itaconic acid production), MEcoli_ref_2 + pl_paba (PABA production) and MEcoli_ref_2 + pl_empty (empty vector, negative control) were inoculated in a 20 ml volume of M9 medium supplemented with 500 mM methanol, 0.1 g l^−1^ yeast extract and chloramphenicol in baffled 100 ml shake flasks. The cultures were incubated at 37 °C, 220 r.p.m., 25 mm throw. In the exponential growth phase, eight (three for pl_empty) replicate 20 ml cultures in M9 medium containing 500 mM methanol were inoculated to an initial OD_600_ of 0.05. At OD_600_ ≈ 1.2 (pl_lac), OD_600_ ≈ 1.0 (pl_ita), OD_600_ ≈ 2.4 (pl_paba) and OD_600_ ≈ 1.1 (pl_empty), production was induced by transferring the cells to fresh medium containing 500 nM aTc (Cayman Chemical). For the transfer, the cultures were centrifuged for 7 min at 3,220 *g*, the supernatant discarded and the cells resuspended in fresh medium. Subsequently, the cultures were incubated in the dark at 37 °C, 180 r.p.m, 50 mm throw. In parallel, eight sterile shake flasks containing medium (20 ml) were incubated to determine the methanol evaporation for calculation of the product yields. Culture samples were collected after: 0, 8, 16, 24, 32 and 40 h (pl_lac); 0, 17, 25, 75 and 115 h (pl_ita); 0, 10.5, 17, 24, 40, 53 and 76.5 h (pl_paba); and 0, 8, 16, 24, 32, 40 and 68 h (pl_empty). The samples were centrifuged for 5 min at 20,000 *g*, 4 °C, and the supernatants were stored at −80 °C until further analysis.

### Production of PHB

The cryogenic glycerol stocks of MEcoli_ref_2 + pl_phb and MEcoli_ref_2 + pl_empty were inoculated as described above. In the exponential phase, three replicate 20 ml cultures in M9 medium containing 500 mM methanol and chloramphenicol were inoculated from the pre-culture. For the transfer, the number of cells required for an initial OD_600_ of 0.05 were centrifuged for 2 min at 5,000 *g* and the supernatant discarded. Subsequently, the pellet was resuspended in a 900 µl volume of M9 medium supplemented with 500 mM methanol and chloramphenicol, centrifuged again for 2 min at 5,000 *g* and the supernatant discarded. The remaining pellet was resuspended in the final medium. The cultures were incubated at 37 °C, 220 r.p.m., 25 mm throw. At OD_600_ ≈ 0.6 (pl_phb) or OD_600_ ≈ 0.9 (pl_empty), production was induced via the addition of 2 µM aTc and the cultures were subsequently incubated in the dark. Cultures were harvested after 74 h (pl_phb) or 69 h (pl_empty). For the harvest, the cultures were centrifuged for 5 min at 11,000 *g* and the supernatant discarded. To remove any residual salt from the cells, the pellet was washed with ultrapure water (50 ml), centrifuged again for 5 min at 11,000 *g* and the supernatant discarded.

### Production of ^13^C-labelled lactic acid

The same protocol as described above to produce PHB was followed, except that MEcoli_ref_2 transformed with pl_lac was grown in M9 medium supplemented with 500 mM ^13^C methanol (99% atomic purity, EURISOTOP).

### Lactic acid and itaconic acid derivatization

The organic acids in the supernatant collected from the cultivation were derivatized together with 200 µM propionate as internal standard according to a modified version of a previously reported protocol^104^. The supernatant samples and the internal standard were mixed and diluted 20-fold in 50% (v/v) acetonitrile. Organic acids were derivatized via the addition of 34 mM 3-nitrophenylhydrazine and 21 mM *N*-(3-dimethylaminopropyl)-*N*′-ethylcarbodiimide, followed by incubation for 1 h at 40 °C, with continuous shaking. The reaction was quenched by the addition of 0.25 volumes of 0.1% (v/v) trifluoroacetic acid and diluted tenfold in 50% (v/v) acetonitrile for LC/MS measurement.

### PHB extraction, depolymerization and derivatization

Cell pellets were dried by lyophilization overnight, weighed and transferred to an air-tight glass tube. A 2 ml sample of 3% (v/v) H_2_SO_4_ in methanol containing 200 µg ml^−1^ benzoic acid as the internal standard and a 2 ml volume of chloroform were added to the pellet. PHB standards were prepared in chloroform and mixed 1:1 (v/v) with 3% (v/v) H_2_SO_4_ in methanol containing benzoic acid. For PHB extraction, depolymerization by methanolysis, and derivatization, samples were incubated for 2.5 h in a boiling water bath. To initiate phase separation, MilliQ ultrapure water (1 ml) was added to the solution and the samples were subsequently mixed gently for 10 min, followed by phase separation for 10 min. The aqueous phase (upper) was discarded and the organic phase (lower) was used for GC analysis. Note that different amounts of biomass were sampled for the negative control and the pl_phb strain. To account for this, the GC traces were scaled accordingly.

### Quantification of lactic acid and itaconic acid via LC/MS

The 3-nitrophenylhydrazone derivatives of lactic and itaconic acid were analysed using UPLC (UPLC Ultimate 3000, Thermo Fisher Scientific) with a C18 column with isobutyl side chains and tetramethylsilane end capping (Kinetex Core-Shell Technology XB-C18: 2.1 × 50 mm, 1.7 μm particle size, 100 Å pore size, Phenomenex) coupled to a hybrid quadrupole-orbitrap mass spectrometer (Q Exactive Plus, Thermo Fisher Scientific). Solvents were 0.1% (v/v) formic acid in ultrapure water (solvent A) and in acetonitrile (solvent B)^104^. To separate the metabolites, the following gradient was used for elution at a constant flow rate of 500 μl min^−1^: 98% solvent A linearly decreased to 5% in 3 min, then held at 5% A for 2 min, then linearly increased to 98% in 0.3 min and held at 98% for a final 2 min. Fourier-transform MS was performed in negative mode with a spray voltage of −3.0 kV, a capillary temperature of 275 °C, an S-lens radiofrequency level of 50, an auxiliary gas flow rate of 20 a.u. and an auxiliary gas heater temperature of 350 °C. Mass spectra were recorded as centroids at a resolution of 35,000 at an *m*/*z* of 200 with a mass range of 150–1,000 *m*/*z* and a scan rate of ~4 Hz in full scan mode. Of each sample, 2 μl was injected. Lactic and itaconic acid were quantified using pure external standards of sodium DL-lactate (Sigma-Aldrich) and itaconic acid (Chemie Brunschwig), respectively, derivatized with internal standard as described above.

LC/MS results were analysed using the eMZed framework (emzed.ethz.ch)^105^. Metabolite peaks were extracted via a targeted approach using commercial standards to define retention time–*m*/*z* peak windows applying an *m*/*z* tolerance of 5 ppm. Integration was performed using trapezoid integration.

### Quantification of PABA via LC/MS

Samples were diluted tenfold in ultrapure water, filtered through a 0.22 µm cellulose acetate filter and diluted eightfold for the initial conditions used for LC/MS. PABA was quantified using UPLC (UPLC Ultimate 3000) equipped with an ethylene-bridged hybrid amide UPLC column (Aquity UHPLC BEH Amide: 130 Å; 1.7 µm; 100 × 2.1 mm; Waters) coupled to the hybrid quadrupole-orbitrap mass spectrometer (Q Exactive Plus). The solvent system consisted of buffer containing 10 mM ammonium formate and 46.7 mM formic acid in ultrapure water mixed 50:50 (v/v) with acetonitrile (solvent A) and 90.50:4.75:4.75 (v/v/v acetonitrile/buffer/methanol) (solvent B). To separate the metabolites, the following gradient was used for elution at a constant flow rate of 500 μl min^−1^: 15.7% solvent A for 1.5 min, linearly increased to 94.7% A over 4 min, held constant at 94.7% A for 2 min, linearly decreased to 15.7% A over 0.5 min and then held at 15.7% A for 2 min (ref. ^106^). The settings for the MS part of the method were Fourier-transform MS in positive mode with a spray voltage of 3.0 kV, a capillary temperature of 275 °C, an S-lens radiofrequency level of 50, an auxiliary gas flow rate of 20 a.u. and an auxiliary gas heater temperature of 350 °C. Mass spectra were recorded as centroids at a resolution of 70,000 at an *m*/*z* of 200 with a mass range of 70–800 *m*/*z* and a scan rate of ~4 Hz in full scan mode. For each sample, a 4 µl volume was injected. LC/MS results were analysed^105^ as described above for lactic and itaconic acid.

### Quantification of PHB by GC-FID

Methanolysed PHB (methyl 3-hydroxybutanoate) was analysed via GC (GC 6850, Agilent Technologies) equipped with a 7683B Series injector coupled to an FID. A DB-WAX column (15 m × 0.32 mm × 0.50 μm; Agilent Technologies) was used for metabolite separation with helium as the carrier gas at a column flow of 1.8 ml min^−1^. The following temperature gradient was applied: 90 °C for 1 min, 1.75 min from 90 to 230 °C, 3 min at 230 °C, 1.27 min to 90 °C and then 1 min at 90 °C. For each sample, a 1 μl volume was injected. The split ratio was 2.0 and the detector temperature was 270 °C. Peaks were confirmed using standards. Benzoic acid was used as the internal standard to correct for methodological variation.

### Quantification of methanol by HPLC

Methanol in samples producing lactic acid were quantified via UPLC (UPLC Ultimate 3000) equipped with a Rezex ROA UPLC column (Rezex ROA-Organic Acid H+ (8%): 300 × 7.8 mm; Phenomenex) coupled to a refractive index detector (RefractoMax 521, Thermo Fisher Scientific). As the solvent, 2.5 mM sulfuric acid in ultrapure water was used. To separate the metabolites, a flow rate of 600 μl min^−1^ was used. Peaks were identified and the peak areas were quantified using Chromeleon 7 software (Thermo Fisher Scientific) on the basis of an external standard curve of pure methanol (Sigma-Aldrich).

### Quantification of methanol by GC-FID

Methanol in the itaconic acid and PABA samples was quantified using GC (GC 6850) equipped with a 7683B Series injector coupled to an FID. A DB-WAX column (15 m × 0.32 mm × 0.50 μm; Agilent Technologies) was used for metabolite separation with helium as the carrier gas at a column flow rate of 2.1 ml min^−1^. The following temperature gradient was applied: 35 °C for 0.5 min, 1.625 min from 35 to 230 °C, 3 min at 230 °C, 1.625 min to 90 °C and then 0.5 min at 90 °C. For each sample, a 0.5 µl volume was injected. The split ratio was 10.0 and the detector temperature was 270 °C. Peaks were confirmed using standards. For the measurements, the samples were diluted 100-fold in 2 mM 1-butanol, which was used as the internal standard to correct for technical fluctuations in the measured values.

### Fed-batch biomass and itaconic acid production in bioreactor

Cryogenic glycerol stocks of MEcoli_ref_2 and MEcoli_ref_2 + pl_ita (itaconic acid production) were inoculated into a 20 ml volume of M9 medium supplemented with 500 mM methanol in 100 ml baffled shake flasks and incubated at 37 °C, 220 r.p.m., 25 mm throw. The cultures were diluted three times during exponential growth, first twice into M9 medium (20 ml) and then into two parallel cultures (200 ml) in one litre baffled shake flasks. For MEcoli_ref_2 + pl_ita, the first pre-culture was supplemented with 0.1 g l^−1^ yeast extract. The bioreactor, prefilled with M9 medium supplemented with 750 mM methanol at 37 °C (one litre), was inoculated with the entire two (200 ml) pre-cultures to a starting OD_600_ of 0.9 for MEcoli_ref_2 and 1.4 for MEcoli_ref_2 + pl_ita. Fed-batch experiments were conducted at 37 °C in a 3.6 litre Labfors 5 Lux bioreactor (Infors) equipped with pH, oxygen partial pressure ($p_{{\mathrm{O}}_2}$) and antifoam sensors. The pH was maintained at 7.0 via the automated addition of 4 M NH_3_. The $p_{{\mathrm{O}}_2}$ was set to 40% saturation during the growth phase, and for itaconic acid it was changed to 50% before induction. Oxygenation was under the control of a serial cascade, first varying the air flow rate from 0.05 to 2 l min^−1^, and subsequently with the stir rate between 200 and 1,200 r.p.m. Antifoam agents were added automatically if foam was detected (MEcoli_ref_2: 10% (v/v) antifoam C and 10% (v/v) antifoam Y-30; MEcoli_ref_2 + pl_ita: 10% (v/v) antifoam L-30). In the case of excessive foam formation, antifoam was added manually to the culture. The OD_600_ value and methanol concentration of the culture were monitored through manual sampling. Methanol was quantified using GC-FID and kept between 500 and 1,000 mM by constant feeding of pure methanol at manually set rates depending on the growth stage of the culture. During growth, the culture was manually supplemented with different medium components to maintain exponential growth. For a detailed overview of the fermentation parameters over time, see Supplementary Data Table 15. To assess the biomass production (cell dry weight per volume) after incubation of MEcoli_ref_2, the culture (10 ml) was sampled in ten replicates and transferred to weighed 15 ml conical tubes. Subsequently, samples were centrifuged for 10 min at 3,220 *g* and the supernatant discarded. Cell pellets were suspended in ultrapure water and centrifuged for 10 min at 3,220 *g*. The supernatant was discarded again and the pellets snap frozen using liquid nitrogen. Frozen pellets were lyophilized overnight and weighed after complete drying. The final cell dry weight per volume was calculated from the weight difference between an empty tube and the conical tube with the pellet. For MEcoli_ref_2 + pl_ita, itaconic acid production was induced by the addition of 500 nM aTc at an OD_600_ of 46. Culture samples were collected regularly, centrifuged for 1 min at 11,000 *g* and the supernatants stored at −20 °C for further analysis.

### Reporting summary

Further information on research design is available in the Nature Portfolio Reporting Summary linked to this article.

### Supplementary Information

Reporting Summary

Supplementary Tables Supplementary Data Tables 1–16.

Supplementary Data 1 Plasmid map of pl_lac.

Supplementary Data 2 Plasmid map of pl_empty.

Supplementary Data 3 Plasmid map of pl_phb.

Supplementary Data 4 Plasmid map of pl_ita.

Supplementary Data 5 Plasmid map of pl_paba.

Supplementary Data 6 Plasmid map of pET16_10His_Mdh_H165N.

Supplementary Data 7 Plasmid map of pET16_10His_Mdh_H165N_F279I.

Supplementary Data 8 Plasmid map of pET16_10His_Gnd.

Supplementary Data 9 Plasmid map of pET16_10His_Gnd_E282*.

Supplementary Data 10 Plasmid map of pET16_10His_PykA.

Supplementary Data 11 Plasmid map of pET16_10His_PykA_Q266K.

Supplementary Data 12 Plasmid map of pET16_10His_PykF.

Supplementary Data 13 Plasmid map of pET16_10His_PykF(L303H).

Supplementary Data 14 List of R and Python versions as well as all packages used.

### Source data

Source Data Fig. 2 Statistical source data.

Source Data Fig. 3 Statistical source data.

Source Data Fig. 4 Statistical source data.

Source Data Extended Data Fig. 1 Statistical source data.

Source Data Extended Data Fig. 2 Statistical source data.

Source Data Extended Data Fig. 3 Statistical source data.

Source Data Extended Data Fig. 4 Statistical source data.

Source Data Extended Data Fig. 5 Statistical source data.

## Data Availability

Data that support the findings of this work are available within the paper, Supplementary Information and Source Data files or from the authors upon reasonable request. The *E. coli* core model is accessible from the BIGG FBA model database (http://bigg.ucsd.edu/models/e_coli_core). Genome resequencing raw files are available from the Sequence Read Archive with BioProject ID PRJNA943419. The accession numbers of samples used for genome sequencing are listed in Supplementary Data Table 11. The *E. coli* BW25113 genome used as template for genome assembly is available at GenBank under accession CP009273. Proteomics data have been deposited to the ProteomeXchange Consortium via the PRIDE (http://www.ebi.ac.uk/pride) partner repository with the dataset identifier PXD046243. Source data are provided with this paper.

## Code availability

Executable scripts and associated data for the generation of all figures and for pFBA analysis are available via the ETH GitLab repository at https://gitlab.ethz.ch/mreiter/methylotrophic_ecoli_bioproduction/ (ref. ^107^). A complete list of software and packages used is provided in Supplementary Data 14.
