# Supplementary material for: A synthetic methylotrophic Escherichia coli as a chassis for bioproduction from methanol
Source: Nat Catal. 2024 Apr 23;7(5):560–73. doi: 10.1038/s41929-024-01137-0 (PMC11136667; doi:10.1038/s41929-024-01137-0)
Supplement: Supplementary file 1 — Reporting Summary [file 41929_2024_1137_MOESM1_ESM.pdf]

nature portfolio

Corresponding author(s): Michael A. Reiter, Julia A. Vorholt

Last updated by author(s): Feb 8, 2024

Reporting Summary

Nature Portfolio wishes to improve the reproducibility of the work that we publish. This form provides structure for consistency and transparency in reporting. For further information on Nature Portfolio policies, see our Editorial Policies and the Editorial Policy Checklist.

Statistics

For all statistical analyses, confirm that the following items are present in the figure legend, table legend, main text, or Methods section.

n/a	Confirmed	
☐	☒	The exact sample size (n) for each experimental group/condition, given as a discrete number and unit of measurement
☐	☒	A statement on whether measurements were taken from distinct samples or whether the same sample was measured repeatedly
☐	☒	The statistical test(s) used AND whether they are one- or two-sided<br>*Only common tests should be described solely by name; describe more complex techniques in the Methods section.*
☒	☐	A description of all covariates tested
☐	☒	A description of any assumptions or corrections, such as tests of normality and adjustment for multiple comparisons
☒	☐	A full description of the statistical parameters including central tendency (e.g. means) or other basic estimates (e.g. regression coefficient) AND variation (e.g. standard deviation) or associated estimates of uncertainty (e.g. confidence intervals)
☐	☒	For null hypothesis testing, the test statistic (e.g. F, t, r) with confidence intervals, effect sizes, degrees of freedom and P value noted<br>*Give P values as exact values whenever suitable.*
☒	☐	For Bayesian analysis, information on the choice of priors and Markov chain Monte Carlo settings
☒	☐	For hierarchical and complex designs, identification of the appropriate level for tests and full reporting of outcomes
☒	☐	Estimates of effect sizes (e.g. Cohen's d, Pearson's r), indicating how they were calculated

Our web collection on statistics for biologists contains articles on many of the points above.

Software and code

Policy information about availability of computer code

Data collection	pFBA was conducted using the cobrapy framework (0.17.1) (Ebrahim, A. et al. COBRApy: COnstraints-Based Reconstruction and Analysis for Python. BMC Syst. Biol. 7, 74 (2013)) under Python (3.7.13).
Data analysis	Growth rates were determined with Dashing Growth Curves (Reiter et al., Dashing Growth Curves – a web application for rapid and interactive analysis of microbial growth curves. 2022.12.16.520708 Preprint at https://doi.org/10.1101/2022.12.16.520708 (2022)).<br>Analysis of Illumina sequencing data was performed with the BBMap (v.38.95) clumpify function (filtering of raw reads for optical and PCR duplicates), and alignment was carried out with Breseq (v.0.36.0) (Deatheridge et al., Identification of Mutations in Laboratory-Evolved Microbes from Next-Generation Sequencing Data Using breseq. in Engineering and Analyzing Multicellular Systems (eds. Sun, L. & Shou, W.) vol. 1151 165–188 (Springer New York, 2014)).<br>LC-MS results were analyzed using the emzed framework (emzed.ethz.ch, version 3) (Kiefer et al., eMZed: an open source framework in Python for rapid and interactive development of LC/MS data analysis workflows. Bioinformatics 29, 963–964 (2013)).<br>Executable scripts and associated data for the generation of all figures and for pFBA analysis are available from the ETH GitLab (https://gitlab.ethz.ch/mreiter/methylotrophic_ecoli_bioproduction/) A complete list of software and packages used is provided in Supplementary Data 14.

For manuscripts utilizing custom algorithms or software that are central to the research but not yet described in published literature, software must be made available to editors and reviewers. We strongly encourage code deposition in a community repository (e.g. GitHub). See the Nature Portfolio guidelines for submitting code & software for further information.

Data

Policy information about availability of data

All manuscripts must include a data availability statement. This statement should provide the following information, where applicable:

- Accession codes, unique identifiers, or web links for publicly available datasets
- A description of any restrictions on data availability
- For clinical datasets or third party data, please ensure that the statement adheres to our policy

Data supporting the findings of this work are available within the paper and its Supplementary as well as Source Data files or from the authors upon reasonable request. The E. coli core model is accessible from the BIGG FBA model database (http://bigg.ucsd.edu/models/e_coli_core). Genome resequencing raw files are available from the sequence read archive (SRA) with BioProject ID PRJNA943419. The accession numbers of samples used for genome sequencing are listed in Supplementary Data 16. The E. coli BW25113 genome used as template for genome assembly is available at GenBank under accession CP009273. Proteomics data have been deposited to the ProteomeXchange Consortium via the PRIDE (http://www.ebi.ac.uk/pride) partner repository with the data set identifier PXD046243.

Research involving human participants, their data, or biological material

Policy information about studies with human participants or human data. See also policy information about sex, gender (identity/presentation), and sexual orientation and race, ethnicity and racism.

Reporting on sex and gender	Not applicable.
Reporting on race, ethnicity, or other socially relevant groupings	Not applicable.
Population characteristics	Not applicable.
Recruitment	Not applicable.
Ethics oversight	Not applicable.

Note that full information on the approval of the study protocol must also be provided in the manuscript.

Field-specific reporting

Please select the one below that is the best fit for your research. If you are not sure, read the appropriate sections before making your selection.

☒ Life sciences ☐ Behavioural & social sciences ☐ Ecological, evolutionary & environmental sciences

For a reference copy of the document with all sections, see nature.com/documents/nr-reporting-summary-flat.pdf

Life sciences study design

All studies must disclose on these points even when the disclosure is negative.

Sample size	Sample sizes were chosen to reflect the state of the art in the field, see for example comparable studies (e.g. https://doi.org/10.1016/j.cell.2020.07.010; https://doi.org/10.1038/s41467-020-19235-5). Besides that, no predetermination of the sample sizes was conducted. The presented data and associated standard deviations demonstrate the number of samples to be sufficient for the statistical comparisons performed.
Data exclusions	No data was excluded from analysis.
Replication	All bioproduction experiments in shake flasks were conducted a second time independently, yielding results comparable to those presented in the paper.
Randomization	As no comparisons between different treatments are made, no randomization was necessary.
Blinding	No blinding was conducted. All experiments included appropriate controls that confirmed assay results.

Reporting for specific materials, systems and methods

We require information from authors about some types of materials, experimental systems and methods used in many studies. Here, indicate whether each material, system or method listed is relevant to your study. If you are not sure if a list item applies to your research, read the appropriate section before selecting a response.

Materials & experimental systems

n/a	Involved in the study
☒	☐ Antibodies
☒	☐ Eukaryotic cell lines
☒	☐ Palaeontology and archaeology
☒	☐ Animals and other organisms
☒	☐ Clinical data
☒	☐ Dual use research of concern
☒	☐ Plants

Methods

n/a	Involved in the study
☒	☐ ChIP-seq
☒	☐ Flow cytometry
☒	☐ MRI-based neuroimaging
